# ZFP57 recognizes multiple and closely spaced sequence motif variants to maintain repressive epigenetic marks in mouse embryonic stem cells

**DOI:** 10.1093/nar/gkv1059

**Published:** 2015-10-19

**Authors:** Zahra Anvar, Marco Cammisa, Vincenzo Riso, Ilaria Baglivo, Harpreet Kukreja, Angela Sparago, Michael Girardot, Shraddha Lad, Italia De Feis, Flavia Cerrato, Claudia Angelini, Robert Feil, Paolo V. Pedone, Giovanna Grimaldi, Andrea Riccio

**Affiliations:** 1 Institute of Genetics and Biophysics ‘A. Buzzati-Traverso’, CNR, 80131 Naples, Italy; 2 Department of Environmental, Biological and Pharmaceutical Sciences and Technologies, Second University of Naples, 81100 Caserta, Italy; 3 Institute of Molecular Genetics (IGMM), CNRS UMR5535 and University of Montpellier, 1919 route de Mende, 34293 Montpellier, France; 4 Istituto per le Applicazioni del Calcolo ‘Mauro Picone’ (IAC), CNR, 80131 Naples, Italy; 5 Ceinge Biotecnologie Avanzate s.c.a.r.l., 80145 Naples, Italy

## Abstract

Imprinting Control Regions (ICRs) need to maintain their parental allele-specific DNA methylation during early embryogenesis despite genome-wide demethylation and subsequent *de novo* methylation. ZFP57 and KAP1 are both required for maintaining the repressive DNA methylation and H3-lysine-9-trimethylation (H3K9me3) at ICRs. *In vitro*, ZFP57 binds a specific hexanucleotide motif that is enriched at its genomic binding sites. We now demonstrate in mouse embryonic stem cells (ESCs) that SNPs disrupting closely-spaced hexanucleotide motifs are associated with lack of ZFP57 binding and H3K9me3 enrichment. Through a transgenic approach in mouse ESCs, we further demonstrate that an ICR fragment containing three ZFP57 motif sequences recapitulates the original methylated or unmethylated status when integrated into the genome at an ectopic position. Mutation of *Zfp57* or the hexanucleotide motifs led to loss of ZFP57 binding and DNA methylation of the transgene. Finally, we identified a sequence variant of the hexanucleotide motif that interacts with ZFP57 both *in vivo* and *in vitro*. The presence of multiple and closely located copies of ZFP57 motif variants emerges as a distinct characteristic that is required for the faithful maintenance of repressive epigenetic marks at ICRs and other ZFP57 binding sites.

## INTRODUCTION

Reprogramming of DNA methylation in early mammalian embryogenesis is a complex process involving a fine balance of methylating and demethylating activities (1). DNA methylation is acquired in oocytes and sperm following different patterns (2). Most of the methylated CpGs established in the gametes are lost in the zygote and during the first cell divisions of the embryo, when cells acquire pluripotency (3,4). This is followed by acquisition of a new methylation landscape during implantation. CpG-rich regions, also known as CpG islands (CGIs), are generally resistant to methylation. About 7% of all CGIs acquire DNA methylation in oocytes, but most of these are demethylated in the blastocyst (3). Despite this genome-wide loss of methylation, a number of sites are maintained methylated in early embryogenesis (2,3). These include the imprinting control regions (ICRs) that are required for the gamete-of-origin-dependent expression of the imprinted genes (5–7). These regions are methylated in the oocyte or sperm (germline-Differentially Methylated Regions, gDMRs) and are maintained methylated on the maternal or paternal allele throughout development (ICRs are demethylated only in primordial germ cells), despite the fact that the majority of them are CpG-rich and correspond to promoters (8). Along with DNA methylation, differential histone modifications are faithfully reproduced at ICRs through cell division, with H3K9me3 and H4K20me3 associated with the DNA methylated allele and H3K4me3 associated with the non-methylated allele (9).

Diverse evidence indicates that the sequence context plays a fundamental role in the determination of the epigenome (10–12). High CpG content is inversely correlated with CpG methylation, likely as a consequence of the binding of zinc finger CxxC domain-containing proteins, such as CFP1 and KDM2A that recognize non-methylated DNA (13). Also, mutation of transcription factor binding sites, such as those of CTCF and SP1, results in increased DNA methylation at the target regions (10,14–16).

Other evidence indicates that factors interacting with methylated DNA contribute to shaping the correct methylation status of the genome as well. Three structural families of methyl–DNA binding proteins (MBPs) with differential recognition of methylated CpGs (mCpG) have been described: mCpG binding domain (MBD), SET and RING finger- associated (SRA) domain and zinc finger (ZFP) (17,18). Although some varying sequence preferences outside of the core mCpG site have been reported for the different MBDs, *in vivo* studies have confirmed that 5-mC is the primary determinant for the genome-wide binding patterns of these proteins and enrichment of binding is correlated with local higher than average DNA methylation density. The SRA domain-containing proteins, including UHRF1, recognize the hemimethylated CpGs that are transiently generated during DNA replication. Differently from the other groups, the ZFPs recognise mCpG within longer specific DNA sequences. This family includes KAISO, ZTB4 and ZBTB38, containing a BTB/POZ protein–protein interaction domain (19); and ZFP57 belonging to the family of Kruppel-associated box (KRAB) domain containing ZFPs (20). Both BTB/POZ and KRAB ZFPs act as chromatin-modulating transcriptional repressors but recruit different chromatin remodelling complexes (19,20).

Loss of the zinc-finger protein ZFP57 or its co-repressor KAP1 (aka Tif1-β or Trim28) results in loss of DNA methylation and H3K9me3 at ICRs in mouse embryos and embryonic stem cells (ESCs), and loss of function of ZFP57 is associated with hypomethylation at multiple imprinted loci and with transient neonatal diabetes in humans (21–25). ZFP57 interacts with the methylated allele of the ICRs where it recruits KAP1 and other associated factors (22,23). The ZFP57 binding sites are enriched of the TGCCGC hexanucleotide motif, and a ZFP57 protein fragment containing two classical Cys_2_His_2_ domains can bind *in vitro* to this DNA motif if methylated (22,26,27). Despite these findings, it remains unclear what are the sequence determinants for ZFP57 binding *in vivo* and how this influences the maintenance of DNA and repressive histone methylation marks at ICRs.

By monitoring the effect of the SNPs present in ESCs derived from intra-specific mouse hybrids, here we investigate the DNA sequence elements required for ZFP57 binding. We identify multiple motif variants that are associated with binding in ESCs and demonstrate that one of these in addition to TGCCGC is able to bind ZFP57 *in vitro*. We also observe that the motif variant sequences are often present in closely spaced clusters at the ZFP57 target sites and that this configuration is necessary for maintaining H3K9me3 at multiple loci. Furthermore, we show that a fragment of the *Snrpn/Snurf* ICR containing three motif sequences is sufficient to maintain the imprinted methylation if integrated at an ectopic locus, while mutation of the motifs resulted in methylation loss. These findings significantly contribute to the structural definition of the mouse ICRs and increase our understanding on the relationship between the genotype and the epigenotype with repressive function.

## MATERIALS AND METHODS

### Cell lines and cu3lture conditions

Wild type (A3 strain) and *Zfp57* -/- (28,29), hybrid JB1 and BJ1 generated from F1 hybrids derived from JF1 x C57Bl/6 (henceforth B6) and B6 x JF1 crosses, respectively (30) and TC-1 (31) ESC lines were cultured under standard feeder-free conditions on gelatinized tissue culture dishes with media containing DMEM (EuroClone ECM0101L) supplemented with 2-mercaptoethanol, non-essential amino acids, sodium pyruvate, 10% fetal calf serum (A3 and *Zfp57* -/-) or 15% fetal calf serum (JB1, BJ1 and TC-1), and leukemia inhibitory factor (LIF) at 37°C under an atmosphere of 5% CO_2_. The culture medium of TC-1 cells was supplied with 25 μg/ml hygromycin B as described by Feng *et al*. (31).

### Chromatin immunoprecipitation (ChIP)

ChIP for the analysis of ZFP57 and KAP1 binding and H3K9me3 was performed on formaldehyde cross-linked chromatin isolated from cells grown on 10 cm dishes to ∼80% confluency. Briefly, the cells were detached by adding 0.05% trypsin at 37°C for 3 min. Formaldehyde was added to the cells resuspended in Phosphate buffered saline (PBS) at a final concentration of 1% and the cells were incubated at room temperature for 15 min with shaking. The reaction was stopped by addition of glycine to a final concentration of 0.125 M. Approximately 3 × 10^7^ cells were washed twice in ice-cold PBS, centrifuged and resuspended in lysis buffer 1 (50 mM HEPES pH 8, 10 mM NaCl, 1 mM EDTA, 10% Glycerol, 0.5% NP-40 and 0.25% Triton X-100) for 90 min at 4°C. Isolated nuclei were lysed in lysis buffer 2 (10 mM Tris–HCl pH 8.0, 200 mM NaCl, 1 mM EDTA and 0.5 mM EGTA) for 60 min at 4°C. The chromatin was sheared in sonication buffer (10 mM Tris–HCl pH 8.0, 100 mM NaCl, 1 mM EDTA, 0.5 mM EGTA, 0.1% sodium deoxycholate and 0.5% N-lauroylsarcosine) to an average size of 100–400 bp using the S220 Focused-ultrasonicators (Covaris).

For each IP, 100 μg of sonicated chromatin were diluted in a final volume of 600 μl with sonication buffer and pre-cleared with 30 μl protein A/G agarose beads (SantaCruz) for 4 h at 4°C on a rotating wheel. Anti-ZFP57 antibody (8 μg, Abcam ab45341), anti-KAP1 antibody (7 μg, Abcam ab10483) and anti-Histone H3K9me3 (7 μg, Abcam 8898) or rabbit IgG were added to the pre-cleared chromatin and incubated overnight at 4°C on a rotating wheel. Chromatin was precipitated with 30 μl protein A/G agarose beads for 4 h at 4°C with rotation. The beads were then washed five times with 500 μl RIPA buffer (10 mM Tris–HCl pH 8.0, 140 mM NaCl, 1 mM EDTA, 0.5 mM EGTA, 0.1% sodium deoxycholate, 1% Triton X-100and 0.1% SDS) and once with each of the following buffers: WASH buffer (50 mM HEPES, 0.5% sodium deoxycholate, 1% Triton X-100, 1 mM EDTA, 500 mM NaCl and 0.2% NaN3), LiCl buffer (0.25 M LiCl, 0.5% NP-40, 0.5% sodium deoxycholate, 1 mM EDTA and 10 mM Tris pH 8) and TE buffer (10 mM Tris pH 8, 1 mM EDTA). The bound chromatin was eluted in 100 μl TE buffer. Crosslinks were reversed by incubation O/N at 65°C after addition of 1 μl RNAse cocktail (Ambion) and 2 h at 50°C after addition of 2.5 μl SDS 20% + 2.5 μl 20 mg/ml proteinase K (Sigma). The DNA was extracted by using the QIAquick Gel Extraction Kit (Qiagen). Immunoprecipitated or 1% input DNAs were analysed by real-time PCR using SBYRGreen PCR Master Mix (Bio-Rad) on a C1000 Thermal Cycler (Bio-Rad). Each reaction was performed in triplicate and all presented results are representative of experiments performed at least twice. Primers are listed in Supplementary Table S1.

### ChIP-Seq analyses

Two nanograms of DNA from immunoprecipitated and input chromatin were used for Illumina library preparation. Libraries were generated and sequenced at IGA Technology Services (Italy), by using the NuGen Ovation Ultralow Library System v2 Kit and 50 bp single-end sequencing on the Illumina HiSeq2500 platform. Sequence reads were processed by adaptor trimming (Illumina Pipeline Casava 1.8.2) and filtering for low quality reads (Trim Galore) and subjected to quality control (FastQC). Reads were aligned to the *Mus musculus* genome (assembly NCBI37/mm9) using the Bowtie algorithm (32). Mapped reads were normalized to reads per million (RPM) using BEDtools and SAMtools and displayed on the UCSC genome browser. Peaks were defined using the MACS algorithm (33) with *P*-values below 1.0 × 10^−5^.

The allele specificity was assessed by mapping the pooled ZFP57/KAP1 reads against the B6 genome (NCBI37/mm9) and the JF1 genome (built using a Python custom script that takes into consideration the SNP data set from NIG Mouse Genome database (34)) individually and not allowing mismatches (Bowtie option –v 0). Duplicate reads and reads overlapping insertion or deletion were removed. We defined as informative reads (IR) those overlapping SNPs in both genomes and allele specific reads ASR^B6^ and ASR^JF1^ the reads matching the SNPs in the B6 and JF1 genomes, respectively. To take into consideration the mapping bias, the same pool of reads were processed in the same manner but allowing up to three mismatches (Bowtie option –v 3) to define the informative control reads (IR_C_) and the allele-specific control reads (ASR_C_^B6^, ASR_C_^JF1^). We excluded the ZFP57 peaks found in the sex chromosomes (hybrid ESC lines were male) and considered the ZFP57 peaks with IR and IR_C_ ≥ 5 in both cell lines. We calculated for each peak the percentage of allelic specific reads *x* = (ASR^B6^ / (ASR^B6^ + ASR^JF1^)) × 100 and the percentage of allelic specific control reads *j* = (ASR_C_^B6^ / (ASR_C_^B6^ + ASR_C_^JF1^)) × 100. We calculated the average ($\bar j$) of the distribution of *j* values and its standard deviation (σ). With Y = $\bar j$ ± 3σ, we defined the range of bi-allelicity. By taking into consideration the results of both JB1 and BJ1 cell lines, we considered the peaks having ((*x*^JB1^ ∈ Y^JB1^) & (*x*^BJ1^ ∈ Y^BJ1^)) as bi-allelic and the peaks with ((*x*^JB1^ ∉ Y^JB1^) and (*x*^BJ1^ ∉ Y^BJ1^)) as mono-allelic; discordant peaks were discarded. To assign a value to measure monoallelicity we created a scoring system that takes into account the results obtained in both reciprocal cell lines. Combined JB1–BJ1 allelic score ${\rm S} = ((|\bar j^{{\rm JB}1} - x^{{\rm JB}1} | + |\bar j^{{\rm BJ}1} - x^{{\rm BJ}1} |))/2$.

### Allele-specific ChIP

ZFP57 binding and H3K9me3 enrichment on the JF1 and B6 alleles of selected loci was determined by typing the immunoprecipitated DNA for SNPs present between the two parental genomes. Input and immunoprecipitated DNAs were amplified by PCR using primers flanking the SNP in both JB1 and BJ1 ESCs. Primers are listed in Supplementary Table S1. The amplification products were sequenced (PRIMM, Italy) and the ratio between B6-specific and JF1-specific DNAs was determined from the electropherogram.

### Gene transfer experiments

The 477 bp fragment of the *Snrpn/Snurf* ICR (mm9, Chr7:67,149,603–67,150,079) was obtained from the genome of *M. m. domesticus* (strain 129P2) by PCR amplification (Primers Snrpn1For1/Snrpn1Rev1 or Snrpn1For2/Snrpn1Rev3, Supplementary Table S1). The fragment was cloned into the plasmids pCpGfree–vitroBmcs (InvivoGen) and pL1–promoter–1L (a kind gift of D. Schübeler) and verified by DNA sequencing (PRIMM). C > A substitutions were introduced individually into the CpG of the three TGCCGC motif sequences by PCR-mediated site directed mutagenesis (35). Briefly, the procedure consisted in three PCR amplification reactions. In the first two reactions, two sub-fragments of the 477-bp sequence were generated by using a flanking WT primer and an internal mutant primer including the C > A substitution. In the third PCR reaction, the two sub-fragments were combined and used as template to generate a full-length mutant fragment with the flanking WT primers. The same procedure was used to add sequentially the second and third mutations to the fragment containing the first mutation. The fragments with single motif mutations were inserted into pCpGfree–vitroBmcs while the fragment with all three mutant motif sequences was inserted into both pCpGfree–vitroBmcs and pL1–promoter–1L. Primers are listed in Supplementary Table S1.

Prior to transfection, the recombinant pCpGfree–vitroBmcs or the recombinant pL1–promoter–1L plasmids were methylated *in vitro* by using the CpG Methyltransferase *M.Sss*1 (BioLabs). Either 4 μg DNA were incubated with 20 u of enzyme in a total volume of 50 μl or 100 μg DNA with 90 u of enzyme in a total volume of 1 ml, as described (36). In both cases, reactions were performed at 37°C for 4 h in the presence of 160 μM S–adenosylmethionine (SAM).

Gene transfers into cultured ESCs were performed by nucleofection, using the Amaxa Nucleofector 2b device (Lonza) with the specific program A-023 and the Mouse ES Cell Amaxa Nucleofector® Kit VPH-1001. About 2 × 10^6^ wild type (WT) or *Zfp57* -/- ESCs were nucleofected with 2 μg linearized recombinant pCpGfree–vitroBmcs. Cre–RMCE targeting was performed as described (36). Briefly, TC-1 cells were cultured for 15 days in the presence of 25 μg/ml hygromycin B prior to transfection; then 4 × 10^6^ cells were nucleofected with 25 μg recombinant pL1–promoter–1L and 15 μg plC–Cre (a kind gift of D. Schübeler). Selection was started 48 h after nucleofection with 8 μg/ml Blasticidin (Invitrogen) for the transfections of the recombinant pCpGfree–vitroBmcs plasmids and 3 μM Ganciclovir for the Cre–RMCE targeting. Successful integration of the *Snrpn/Snurf* ICR transgenes was verified by PCR amplification.

### Methylation analysis

Genomic DNA (2 μg) was bisulfite-converted with the EpiTect Bisulfite Kit (QIAGEN). The regions of interest were amplified by PCR. The methylation status was studied with three methods: bisulfite sequencing of individual clones, direct bisulfite sequencing and COBRA (Combined Bisulfite Restriction Analysis). For the first method, PCR products were cloned into the TOPO TA cloning vector (Invitrogen) and individual clones isolated and sequenced (PRIMM). DNA sequences were analysed by using the Chromas Lite 2.1.1 (Technelysium) program. Direct bisulfite sequencing was obtained by direct sequencing of the bisulfite-treated and PCR-amplified genomic DNA. COBRA was performed as already described (22). Briefly, the amplified DNA fragments (primers pCGfBmcsF and pCGfBmcsR for the recombinant pCpGfree–vitroBmcs plasmids and TOPL1pBF1 and TOPL1pBR1 for the RMCE system) were radiolabeled (α^32^ P-dCTP) and digested with a restriction enzyme (*Mlu*I or *Hha*I) containing a CpG in its recognition site. Digested fragments were resolved on a non-denaturing PAGE gel. Methylation level was determined by estimating the relative amount of digested and undigested fragments by using the PhoshorImager and ImageQuant software (PerkinElmer). Primers are listed in Supplementary Table S1.

### Protein expression and purification, Electrophoretic mobility shift assay (EMSA)

The DNA fragment encoding the region from Glu87–Ala195 residues of mouse ZFP57 was cloned into the expression vector pMalC2x. The plasmid was used to express mouse ZFP57 fused to the maltose binding protein (MBP) in *E. coli* Bl21. The protein was purified according to the manufacturer's protocol (NEB). For EMSA, 25 pmoles of purified protein were incubated on ice for 10 min with 2.5 pmoles of methylated or unmethylated double-stranded oligonucleotides (sequences reported in Supplementary Table S1) in the presence of 25 mM HEPES pH 7.9, 50 mM KCl, 6.25 mM MgCl_2_, 5% glycerol. After incubation, the mixture was loaded onto a 5% polyacrylamide gel and run in 0.5% TBE (200 V for 75 min). The gels were then stained with SYBR Green (Invitrogen). Competition experiments were performed by using a 5′-FAM (caroxyfluorescein)-labeled double-stranded oligonucleotide containing the 5′-TGCCGC-3′ hexamer (22) as probe and adding unlabelled competitors to the reaction mixture. All gels were imaged with a Typhoon Trio++ scanner (GE Healthcare). Probes are listed in Supplementary Table S1.

### Gene expression analysis

For allele-specific expression analysis, 1 μg of total RNA from cells JB1 and BJ1 ESCs was retrotranscribed by using the QuantiTect Reverse Transcription Kit (Qiagen), according to the protocol of the manufacturer. DNA and cDNA were amplified by PCR (conditions available upon request) in transcribed regions including al least one SNP. The amplification products were sequenced (Eurofins Genomics) over the SNPs and the allele-specific expression was estimated from the ratio between B6-specific and JF1-specific nucleotides in the cDNA electropherogram. Genomic DNA was used as control. DNA sequences were visualised by using SnapGene viewer. Primers are listed in Supplementary Table S1.

## RESULTS

### Allele-specific binding of ZFP57 in mouse ESCs

**Figure 1.**
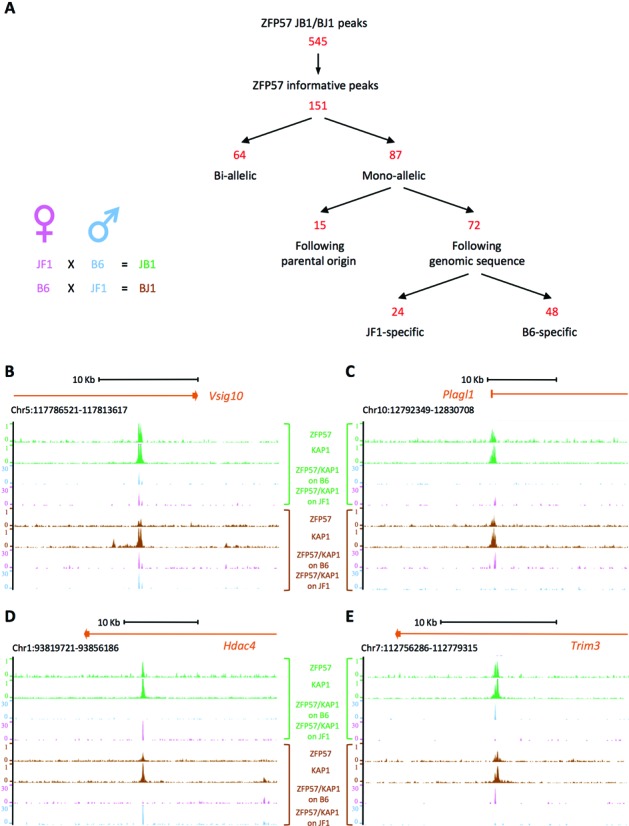
Genome-wide allele-specific analysis of ZFP57 binding. (**A**) Left, the hybrid ESC lines derived from reciprocal crosses between JF1 and B6 mouse strains. Right, strategy used for the identification of allele-specific binding of ZFP57. (**B–E**) Screen shots from the UCSC genome browser describing examples of bi-allelic binding (**B**) and parental origin-specific (**C**), JF1 strain-specific (**D**) and B6 strain-specific (**E**) monoallelic ZFP57 binding as analysed by ChIP-seq. Tracks showing the genomic ZFP57 and KAP1 binding profiles in JB1 (green) and BJ1 (brown) ESCs, and the B6-specific and JF1-specific ZFP57-KAP1 reads (pink if derived from the maternal allele, blue if derived from the paternal allele) obtained in either JB1 and BJ1 ESCs.

We took advantage of the high number of SNPs that are present between the genomes of different sub-species of *Mus musculus* to identify the sequence determinants for ZFP57 binding in cultured cells. In particular, we compared the binding profiles of ZFP57 in the genomes of the Japanese fancy mouse 1 (*Mus musculus molossinus* strain JF1) and *Mus musculus domesticus* strain B6 that differ for >10 million SNPs (34). In order to directly compare the binding capability of both these genomes, we used two lines of mouse ESCs (JB1 and BJ1) generated from F1 hybrids derived from JF1 x B6 and B6 x JF1 crosses, respectively (30). The binding of ZFP57 and KAP1 was determined by ChIP-seq in both JB1 and BJ1 ESCs (Figure 1A). The reads were first mapped against the B6 reference genome by allowing up to two mismatches/read. Peaks were called by using the MACS algorithm (33). Candidate peaks with *P*-values below 1.0 × 10^−5^ were called. 545 ZFP57 precipitation peaks that coincided with KAP1 binding sites and were present in both reciprocal cell lines were identified (Supplementary Table S2). To increase power and have information on the allele-specific binding of the ZFP57–KAP1 complex, we pooled the ZFP57- and KAP1-specific reads. For the analysis of allele-specificity, the combined reads were mapped against the B6 and JF1 genomes individually. We excluded the ZFP57 peaks found in sex chromosomes (hybrid ESC lines were male) and those covered by insufficient number of reads overlapping SNPs (informative reads, IR) in one or both cell lines. ZFP57 peaks with IR equally distributed between the B6 and JF1 genomes in both cell lines (allelic score S ≤ 26, see Methods section for details) were considered bi-allelic, while the peaks with biased distribution (S ≥ 27) were considered mono-allelic (Supplementary Table S2). Discordant peaks between the two cell lines were discarded. The results of this analysis (Supplementary Table S3) produced a list of 151 informative peaks of which 64 bi-allelic (Figure 1A and example in 1B) and 87 mono-allelic sites (Figure 1A, and examples in Figure 1C–E). The mono-allelic sites included 15 peaks corresponding to 14 loci following the parental origin rather than the sequence, being mapped to the JF1 genome only in JB1 and to the B6 genome only in BJ1 (example in Figure 1C). Among these, the 12 loci with the most strongly biased distribution (S > 42.5) corresponded to known ICRs for which ZFP57–KAP1 binding had been described previously (22). Another couple of sites (*Gm16083* and *Kcnk2*) for which the imprinting status was unknown showed moderately a biased distribution (S = 40 and S = 27, respectively) with preferential precipitation of the maternal allele (Supplementary Table S3). These sites are relatively distant from the annotated UCSC genes (>37 Kb from the 3′-end and >90 Kb from the 5′-end of the closest gene). We analysed the allele-specific expression of *Zp3r* that is the closest gene to the ZFP57 site named *Gm16083* (S = 40). Expression was found mono-allelic but B6-specific in both JB1 and BJ1 cell lines, ruling out the imprinted expression of this gene in mouse ESCs (Supplementary Figure S1). Another 72 peaks were mouse strain-specific. Of these, 24 were JF1-specific (Figure 1A and example in Figure 1D) and 48 were B6-specific (Figure 1A and example in Figure 1E) in both the reciprocal crosses. The allele-specific binding of ZFP57 was validated at several of these sites (33 ≤ S ≤ 62) by locus-specific ChIP (Supplementary Figure S2). At these loci, it is likely that one or more SNPs interfered with ZFP57 binding either on the JF1 or the B6 genome. Conversely, the SNPs did not interfere with ZFP57 binding at the sites showing bi-allelic enrichment. In conclusion, this approach provided a list of DNA sequence changes that were permissive and a list of changes that were non-permissive for ZFP57 binding.

### Sequence determinants for ZFP57 binding in mouse ESCs

**Figure 2.**
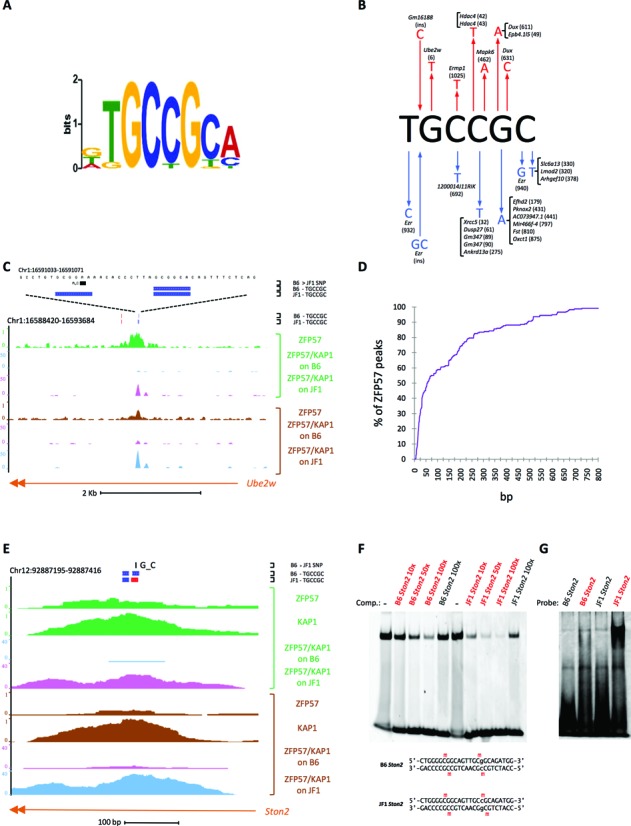
Identification of the sequence determinants for ZFP57 binding. (**A**) Logo of recurrent motifs identified in the binding-permissive relative to the non-permissive alleles by using the MEME algorithm. (**B**) SNPs (arrows pointing outwards) and insertions (arrows pointing inwards) affecting the TGCCGC motif and resulting in loss of ZFP57 binding in either B6 (red) or JF1 (blue) genomes. The IDs of the peaks in which the SNPs are present and the SNP reference numbers (Supplementary Table S4) are indicated. (**C**) Screen shot showing an example of JF1-specific ZFP57 binding associated with a SNP in one of two closely located motifs. (**D**) Empirical cumulative distribution of the distances separating two TGCCGC motifs in the genomic regions covered by ZFP57 peaks. (**E**) Screen shot of the *Ston2* locus showing binding of ZFP57 to a couple of motifs in inverted orientation on the JF1 genome and absence of binding to the motifs with same orientation on the B6 genome. Motif sequences present on the forward strand are in red, those on the reverse strand in blue. (**F–G**) *In vitro* analysis of ZFP57 binding to the B6 and JF1 alleles of *Ston2* as revealed by EMSA either in a competition experiment (**F**) with a probe containing a previously described ZFP57 binding site (22) or directly (**G**) using *Ston2* sequences as probes. For the competition experiment (**F**), a 5′-FAM-labeled double-stranded 20mer oligonucleotide probe was incubated with a protein fragment containing the DNA binding domain of mouse ZFP57 and subjected to PAGE either in absence or presence of 10-, 50- or 100-fold excess of unlabelled oligonucleotides containing the *Ston2* sequence from either the B6 or the JF1 genome in either unmethylated or methylated (red) form. For the analysis of direct binding (**G**), the unlabeled *Ston2* oligonucleotides, in unmethylated or methylated (red) form were used as probes and the gels were stained with SYBR Green. The experiments have been repeated three times with similar results. The B6 and JF1 oligonucleotide sequences are shown below the gel. Comp, competitor. Note that ZFP57 binds with higher affinity the JF1 *Ston2* allele than the B6 *Ston2* allele.

In order to identify the DNA sequence determinants for ZFP57 binding at the loci that show genotype-specific binding, we compared the strings surrounding (± 5 bp) the SNPs of the permissive alleles with the strings of the non-permissive alleles (Supplementary Table S4). We used the MEME suite (37) to look for recurrent motifs in the permissive alleles relative to the non-permissive alleles. The identified logo (E-value = 4.3 × 10^−13^) includes as nucleotides with the maximum likelihood ratio the TGCCGC motif identified by comparing the ZFP57 binding sites in mouse ESCs (22), demonstrating that this sequence is the main determinant of ZFP57 binding *in vivo* (Figure 2A). We observed that more than one third (25/72) of the loci with strain-specific binding was associated with SNPs disrupting one or more copies of this motif in the non-permissive alleles (Figure 2B). In contrast, in only 1/72 loci (*Ston2*) a SNP disrupted the TGCCGC in the permissive alleles. From the analysis of the sequence changes associated with loss of binding, we observed that most of these variants affected the CpG dinucleotide, but some changes including two insertions/deletions also affected all the other conserved positions of the motif (Figure 2B). We also observed that in 21 of the 25 cases with strain-specific binding the SNP disrupted only one out of several motif copies present in the peak region and in only 4 loci the SNPs disrupted all (one or two) motifs present. In addition, in 13/25 loci the SNPs disrupted one or both couples of motifs less than 38 bp apart (example in Figure 2C). From the distribution of the TGCCGC motifs in all ZFP57 peaks, we found that the number of motifs/peak was on average 1.8 ± 2.4 (Supplementary Table S2) and that couples of motifs less than 38 bp apart were present in 44% of the peaks with at least two motifs (236 peaks, see Supplementary Table S5), including 14/18 ICRs (Figure 2D). In summary, these data demonstrate that ZFP57 binding is affected by SNPs occurring in one copy of the several and generally closely associated TGCCGC motif sequences that are present at the target sites.

From the analysis of the loci with evidence for bi-allelic ZFP57 binding, we found that only two of these 64 sites had a SNP between B6 and JF1 within the motif (Supplementary Table S4). In one (*Zfp11*) of these loci, two closely located SNPs disrupted one motif on each of the two alleles, maintaining identical the number (4 copies) in the B6 and JF1 genomes. The other locus (*Arhgap10*) contained a repetitive sequence with many motifs and the SNPs only slightly reduced (19 to 18) their total number on one of the two alleles. Also the motifs of the 20 known ICRs were generally not affected by SNPs. At only one ICR (*Peg13*), one of the five motifs was mutated on the JF1 allele, but this did not abolish ZFP57 binding (data not shown).

We then analysed in further details the only locus (*Ston2*) in which a SNP disrupted the TGCCGC motif in the binding-permissive allele (see above). In this case, a G > C mutation in one of two closely spaced (2–3 bp) motifs changed their relative orientation. Intriguingly, the JF1 allele with the motifs in inverted orientation was associated with binding while the B6 allele with the motifs in the same orientation was not (Figure 2E). We then tested *in vitro* the binding of ZFP57 to these alternative sequences. Specifically, the protein fragment containing the DNA binding domain of ZFP57 was produced in *E. coli* and its interaction with double-stranded oligonucleotides was measured by Electrophoretic Mobility Shift Assay (EMSA). First, we tested if the B6 and JF1 *Ston2* alleles were able to compete with a probe previously shown to be a good binding site for ZFP57 (22). Interestingly, the results demonstrated that the methylated JF1 allele was binding ZFP57 *in vitro* with higher affinity than the methylated B6 allele (Figure 2F). Consistent results were obtained when these two sequences were tested directly as probes (Figure 2G). Thus, efficient binding to the inverted motifs (**JF1 *Ston2***) but poor binding to the directly repeated motifs (**B6 *Ston2***) were observed, suggesting that the binding affinity of ZFP57 to adjacent target sites increases when the motifs are present in inverted orientation, while steric hyndrance inhibits it when the motifs are present in the same orientation. Altogether, these data indicate that interaction of ZFP57 molecules binding to adjacent target sites may affect the binding affinity.

### Epigenetic modifications and gene expression at mono-allelic ZFP57 binding sites

**Figure 3.**
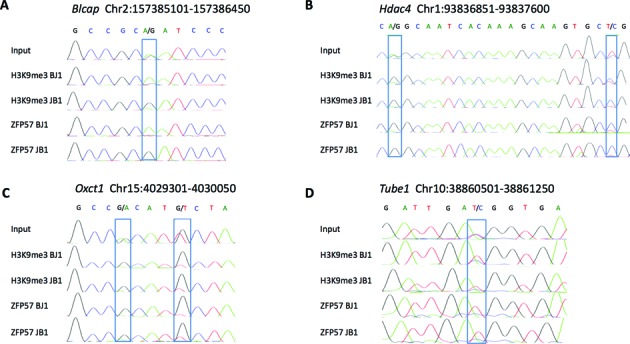
Allele-specific H3K9me3 and ZFP57-binding at loci with mono-allelic ZFP57 binding. DNA sequencing electropherograms showing relative enrichment of B6 and JF1 alleles at the *Blcap* ICR and three loci with strain-specific ZFP57 binding in H3K9me3 and ZFP57 ChIP DNAs obtained in JB1 and BJ1 ESCs. SNPs are boxed. The experiments have been repeated three times with similar results.

To determine the epigenetic modifications of mono-allelic ZFP57 binding sites, we looked at H3K9me3 and CpG methylation, epigenetic marks both known to be associated with ZFP57 binding sites (22). For this type of analysis, we used a locus-specific approach. We analysed the allele-specific enrichment of H3K9me3 of 7 loci showing mono-allelic ZFP57 binding including an ICR (*Blcap/Nnat*) and 6 loci with strain-specific ZFP57 binding. In addition to the *Blcap/Nnat* locus (Figure 3A), 3 further loci (*Hdac4, Oxct1* and *Tube1*) showed higher H3K9me3 enrichment on the parental allele that was bound by ZFP57 (Figure 3B–D). At the remaining three loci (*Pld1, Trim3* and *Ube2w*), H3K9me3 was enriched with similar intensity on the two parental alleles (Supplementary Figure S3). The analysis of DNA methylation was performed by bisulfite sequencing of individual clones on three selected loci showing strain-specific mono-allelic binding of ZFP57 (*Tube1, Nhlrc1, Ube2w*). The results showed that these sites were CpG methylated at variable level but no significant difference was found between the ZFP57 permissive and non-permissive alleles (Supplementary Figure S4). Therefore, allele-specific differences in ZFP57 binding correlate with differences in H3K9me3 enrichment at several non-ICR loci but no effect was observed on the mCpG level of this category of ZFP57 binding sites. To investigate what consequence strain-specific ZFP57 binding had on gene expression, we analysed the allele-specific expression of genes that were located close to strain-specific sites. We selected four genes that were expressed in ESCs and contained SNPs in their exons. Two of these genes (*Dusp27* and *Xrcc5*) showed preferential B6-specific expression and the remaining two (*Derl1* and *Pard3*) had equivalent expression from the B6 and JF1 genomes (Supplementary Figure S5 and Supplementary Table S6). For both *Dusp27* and *Xrcc5*, the preferentially expressed allele was the one binding ZFP57. These data indicate that at some non-ICR loci allele-specific binding of ZFP57 is paralleled by allele-specific gene expression, although the exact role of ZFP57 binding on the expression of these loci remains to be determined.

### Binding of ZFP57 to an ectopic fragment containing multiple methylated sequence motifs

**Figure 4.**
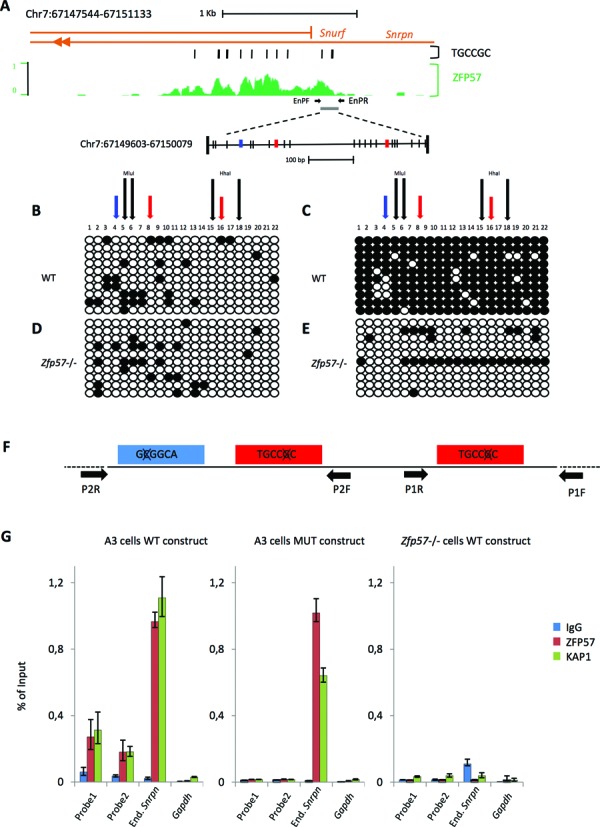
DNA methylation and ZFP57 binding at the 477 bp *Snrpn/Snurf* ICR transgene in WT and *Zfp57* -/- ESCs. (**A**) Screenshot of the UCSC genome browser showing the genomic profile of ZFP57 binding in JB1 ESCs relative to the *Snrpn/Snurf* ICR. The location of TGCCGC motifs and 477 bp fragment used for transgenic analysis is indicated. CpGs are indicated as vertical bars. TGCCGC motif sequences present in the forward strand are in red, those present in the reverse strand are in blue. The primers (EnPF and EnPR) used for the locus-specific ChIP analysis (**G**) of the endogenous *Snrpn* locus are indicated by horizontal arrows. (**B–E**) DNA methylation analysis by bisulfite sequencing of individual clones of the *Snrpn/Snurf* ICR transgene integrated in non-methylated (**B**, **D**) or methylated (**C**, **E**) form into WT (**B**, **C**) or *Zfp57* -/- (**D**, **E**) ESCs. Methylated and unmethylated CpG dinuclotides are represented as open and filled circles, respectively. Black vertical arrows indicate the positions of *Hha*I and *Mlu*I restriction sites used for COBRA (Figure 5A). Red and blue vertical arrows indicate the positions of the motifs on the forward and reverse strand, respectively. One representative ESC clone for each transfection is shown. (**F**) Diagrammatic representation of the TGCCGC motif mutagenesis in the 477 bp ICR transgene. The primers (P1F/P1R and P2F/P2R) used for the ChIP analysis of the 477 bp transgenes (**G**) are shown as horizontal arrows. (**G**) ZFP57 and KAP1 enrichment on the transgenic WT or T-mut fragments after introduction into WT or *Zfp57* -/- ESCs, as determined by ChIP. Two regions (probe 1 and probe 2) of the 477 bp transgenes were analysed, by using the primers P1F/P1R and P2F/P2R, respectively. The endogenous *Snrpn/Snurf* ICR and *Gapdh* were analysed as positive and negative controls, respectively. The results are reported as percentage of input.

Having established that the sequence motif necessary for ZFP57 binding was often present in multiple copies, we sought to test if a DNA fragment containing three motif sequences was sufficient for establishing ZFP57 binding at an ectopic site. To this purpose, a gene transfer approach by stable transfection in cultured ESCs was chosen. The ZFP57 peak region corresponding to the ICR of the *Snrpn/Snurf* locus contains 10 TGCCGC motif sequences (Figure 4A). From this region, we isolated a fragment of 477 bp encompassing the 5′ end of the *Snrpn/Snurf* gene and including three motifs, of which two closer ones and a relatively more distant one (Figure 4A). This fragment was tested for the capacity of binding ZFP57 if introduced into the genome at an ectopic location. Since ZFP57 preferentially binds methylated DNA (22), we first determined if the 477 bp fragment was able to maintain its methylation status when introduced into WT ESCs. Specifically, we integrated the 477 bp fragment in either a methylated or a non-methylated form into the genome of WT ESCs by cell transfection. For *in vitro* methylation of all CpG dinucleotides of the fragment, we used the *SssI* DNA methyltransferase. The DNA methylation level of the integrated transgenes was determined by bisulfite sequencing of individual clones in the transfected cells after 18 days of culturing under selecting conditions. The results demonstrated high levels (91.4%) of CpG methylation of the fragment transfected in the methylated form and low level (12.7%) of methylation of the fragment transfected in the unmethylated form. This indicates that at least after 15 cell cycles the *Snrpn/Snurf* ICR transgene is able to somatically maintain its original methylation status when integrated at an ectopic position in WT ESCs (Figure 4B). We then tested if the maintenance of the DNA methylation status was dependent on ZFP57. To test this hypothesis, we introduced the unmethylated and *in vitro*-methylated 477 bp fragment into *Zfp57* -/- ESCs (29). Relatively low methylation levels (11.4% and 13.6%, respectively) were detected in this case, indicating that the maintenance of methylation on the transgene was dependent on the presence of ZFP57 (Figure 4D–E).

The next step was to investigate if ZFP57 was able to bind the methylated 477 bp fragment when integrated into the ESC genome and if the TGCCGC motif was required for binding. To address this question, we generated a mutant fragment (T-mut) in which the cytosine of the methylated CpG recognized by ZFP57 was mutated into adenine in the three motif sequences (Figure 4F). ZFP57 and KAP1 binding to both WT and mutant fragments was measured by ChIP followed by locus-specific qPCR. The results showed higher ZFP57/KAP1 enrichment on the WT fragment relative to the T-mut fragment after transfection into WT ESCs. Moreover, poor ZFP57/KAP1 binding was observed on the WT fragment when transfected into *Zfp57* -/- ESCs (Figure 4G). We also observed that ZFP57/KAP1 enrichment on the transfected fragment was 4- to 5-fold lower than that on the endogenous *Snrpn* ICR in WT ESCs. This is consistent with the observation that sequences adjacent to the 477 bp fragment also contribute to ZFP57 binding (see Figure 4A). In summary, we were able to reproduce the binding of ZFP57 to a methylated fragment ectopically integrated into ESC genome and demonstrate that this binding is dependent on the TGCCGC motif.

### Role of the ZFP57 target sites in DNA methylation maintenance of the *Snrpn* ICR transgene

**Figure 5.**
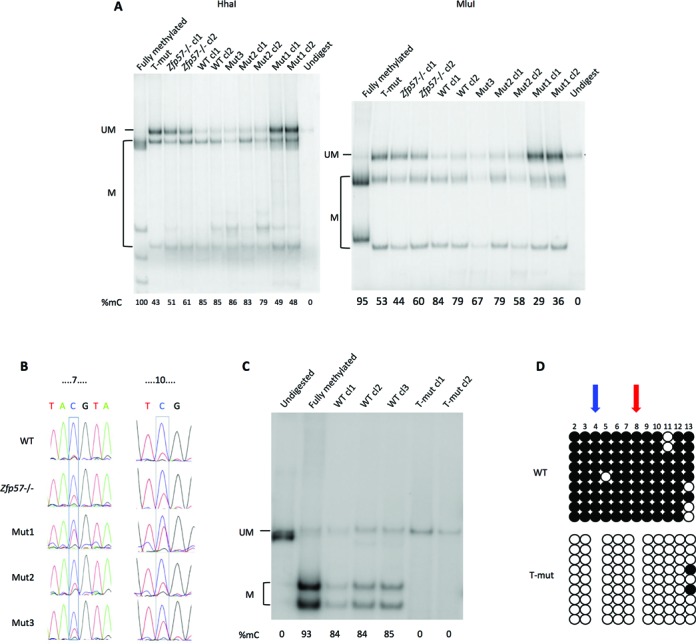
DNA methylation analysis of the WT and mutant *Snrpn/Snurf* ICR transgenes. (**A**) COBRA analysis of the WT, T-mut and single mutant (mut1, mut2 and mut3) fragments after transfection in WT ESCs and of the WT fragment after transfection in *Zfp57* -/- ESCs. Two restriction enzymes (*Hha*I and *Mlu*I) are used. The level of methylation is indicated by the intensity of the higher mobility bands (M) relative to the intensity of the band of the full-length fragment (UM). The *in vitro* fully methylated fragment was used as control and the undigested fragment run as marker. Multiple clones of each construct were analysed. (**B**) Direct bisulfite sequencing of WT fragment introduced into WT or *Zfp57* -/- ESC, and WT, mut1, mut2 and mut3 fragments introduced into WT ESCs. Numbers above the sequence indicate the number of the CpGs analysed, according to Figure 4B. One representative ESC clone for each transfection is shown. (**C**) COBRA analysis of WT and T-mut transgenes in individual clones of WT ESCs in which the WT or T-mut fragments have been introduced into the β*-Globin* locus by RMCE. Multiple clones of each construct were analysed by using the HhaI enzyme. (**D**) DNA methylation analysis by bisulfite sequencing of individual clones of the transgenes described in **C**. One representative ESC clone with the WT transgene and one ESC clone with the T-mut transgene are shown. Location of the TGCCGC motifs is indicated by red and blue arrows if present on the forward or reverse strands, respectively. Experiments were repeated three times with similar results.

After demonstrating that ZFP57 was necessary for DNA methylation maintenance and that the TGCCGC motifs were necessary for ZFP57 binding at the *Snrpn* ICR transgene, we looked if the ZFP57 target sites themeselves were necessary to maintain DNA methylation. For this study, DNA methylation was determined by using the more quantitative COBRA and direct bisulfite sequencing methods. Consistent with the results obtained by bisulfite sequencing of individual clones, the *in vitro* methylated WT fragment was found highly methylated (83.3% ± 2.9) after transfection in WT ESCs, while the same fragment showed lower methylation levels (54% ± 6.9) if transfected into *Zfp57* -/- ESCs (Figure 5A and B). The lower methylation level found in the *Zfp57* -/- ESCs by using the former method (Figure 4E) is likely the result of cloning selection. We then tested the effect of the triple motif mutation (T-mut) and found lower methylation level (48% ± 5.0) as well, demonstrating the importance of the ZFP57 binding sites for DNA methylation maintenance of the *Snrpn/Snurf* ICR transgene. To examine the role of each ZFP57 binding motifs, we introduced constructs carrying individual motif mutations into the cells and determined the effect of each of them on methylation. The results demonstrated that each of the mutant transgenes had lower levels of DNA methylation relative to the WT (Figure 5A and B). However, the results also demonstrated that one of the mutations (mut1) had a stronger effect than the others, with levels of methylation (40.5% ± 8.4) comparable to those observed with the triple mutant and significantly lower than those of the other mutants (74.7% ± 9.8 for mut2 and 76.5% ± 9.5 for mut3).

In the experiments described so far, the WT and mutant *Snrpn* transgenes were randomly integrated into the ESC genome. By using a homologous recombination approach (Cre–RMCE targeting system (31)), we also tested the effect of the motif mutations after targeting the fragments to a specific genomic region (β*-Globin* locus) into WT ESCs. The results obtained by COBRA and bisulfite sequencing of individual clones demonstrated high methylation level (84.3%) on the WT fragment and very low methylation (<5%) on the T-mut fragment, confirming the critical role of the ZFP57 target motifs in DNA methylation maintenance of the *Snrpn/Snurf* ICR (Figure 5C–D).

### Role of binding motif variants

**Figure 6.**
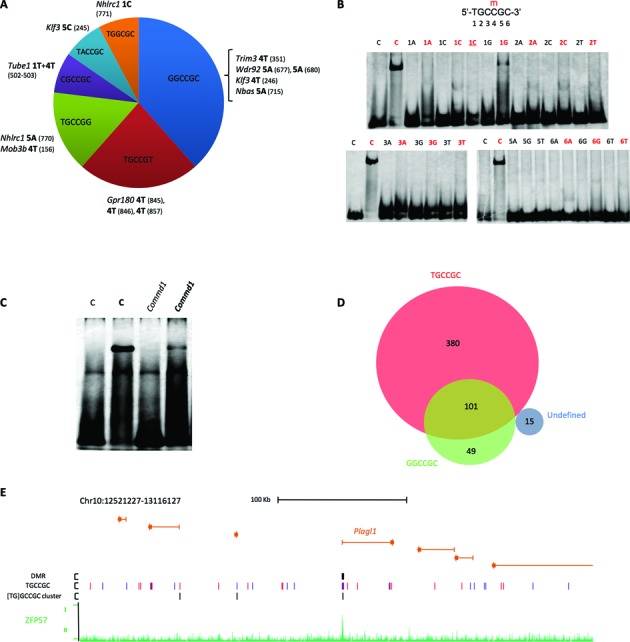
Analysis of the ZFP57 motif variants. (**A**) Sequence motif variants with nucleotide changes found in non binding permissive alleles. The peak regions IDs containing the SNPs are in italics. The base change and its position in the hexamer motif variant are in bold. The SNP IDs are in brackets (Supplementary Table S4). (**B**) *In vitro* analysis of ZFP57 binding to motif variants as determined by Electrophoretic Mobility Shift Assay (EMSA). 20mer double-stranded oligonucleotide probes containing the hexameric sequence represented above (**C**) or single-nucleotide variants (indicated above the lanes) in either unmethylated or methylated (red) form were analysed as described in Figure 2G. The experiments were repeated three times with similar results. **1C** (red), T > C variant with two methylated CpGs. (**C**) *In vitro* analysis of ZFP57 binding to the *Commd1* locus as determined by EMSA. (**D**) Venn diagram of ZFP57 peaks containing TGCCGC and GGCCGC motif variants in the *Mus musculus* genome (assembly NCBI37/mm9). The udefined set does not contain neither TGCCGC or GGCCGC. Note that 2 of the 15 undefined peaks contain the TGCCGC motif in the JF1 genome. (**E**) Clusters of closely located motif variant sequences at ICRs. UCSC screen shot showing the binding profile of ZFP57 at the *Plagl1* locus, and location of the ICR, TGCCGC motifs and [TG]GCCGC clusters of at least two motif sequences less than 38 bp apart.

Mutations in the canonical TGCCGC binding motif do not explain all strain-specific differences in ZFP57 binding. From the analysis of the sequence changes of the loci with sequence-specific binding, we noted that not all possible base substitutions of the TGCCGC motif were found associated with loss of binding in hybrid ESCs (Figure 2B). Indeed, several of the 47 loci with unexplained strain-specific binding had sequences differing from TGCCGC at a single position outside of the CpG in the binding-permissive allele and SNPs in these sequences were associated with loss of binding, suggesting that they may also be recognized by ZFP57 (Figure 6A). We then tested *in vitro* ZFP57 binding to these motif variants. The results demonstrated that, in addition to TGCCGC, the ZFP57 DNA binding domain interacted with the oligonucleotide containing the sequence GGCCGC, in case the CpG was methylated (Figure 6B). All other tested variants showed poor binding in either the CpG-methylated or unmethylated form. We also tested *in vitro* ZFP57 binding to the *Commd1* ICR that has two overlapping GGCCGC sequences in inverted orientation, shows ZFP57 binding in ESCs, but lacks the TGCCGC sequence (Supplementary Figure S6). The results obtained by EMSA demonstrated *in vitro* binding of the ZFP57 protein fragment to *Commd1* if the central CpG was methylated, although the binding efficiency appeared lower than that of the control TGCCGC sequence (Figure 6C). From the analysis of the GGCCGC sequence in the entire mouse reference genome, we found that 28% of all ZFP57 peaks and 77% of those lacking the TGCCGC motif contained this motif variant (Figure 6D). We then recalculated the distribution of the motifs in all genomic ZFP57 peaks by taking into account also the GGCCGC variant and found that the [TG]GCCGC motifs/peak were now 2.4 ± 2.5 on average (Supplementary Table S2) and that motif couples less than 38 bp apart were present in 61% of the peaks with at least two motifs (Supplementary Figure S7 and Supplementary Table S5). Significantly, these peaks now included all ICRs. Overall, we found one cluster of two or more motifs less than 38 bp apart every 2276 bp of genome covered by ZFP57 peaks, but only one cluster every 161914 bp in the entire mouse genome, indicating an enrichment of 71-fold at the ZFP57 binding sites (Figure 6E and Supplementary Table S5). These findings greatly improve the structural definition of the ZFP57 binding sites.

## DISCUSSION

The maintenance of epigenetic states with either repressive or permissive properties at specific loci is a key process in early embryonic development. This study identifies CpG-rich DNA sequence elements that are required for the somatic maintenance of repressive DNA and H3K9me3 methylation in mouse ESCs. Our data complement extensive earlier work indicating that other *cis*-acting elements are necessary for maintaining the DNA methylation-free status of CpG-rich regions (12). We first defined the precise DNA sequence determinants for ZFP57 binding in mouse ESCs and demonstrate that these sequences are necessary for the faithful maintenance of H3K9me3 at several loci. By using a transgenic approach, we found that the ZFP57 target motifs are strictly required for maintaining CpG methylation at an ICR. We further demonstrate that ZFP57 can recognize multiple motif sequence variants both *in vivo* and *in vitro*. Our combined findings evokes a mechanistic model in which clustering of closely spaced motif sequences is required for efficient ZFP57 recruitment and binding and for the maintenance of repressive DNA methylation and H3K9me3.

It has been demonstrated previously that the TGCCGC motif is enriched at ZFP57 binding sites in mouse ESCs, and that a protein fragment containing two ZFP57 zinc fingers is able to interact *in vitro* with a double-stranded oligonucleotide containing the CpG-methylated version of this canonical motif (22,26). Our current study shows that SNPs that alter the TGCCGC hexanucleotide sequence are associated with a lack of ZFP57 binding. They provide a direct demonstration that this motif is strictly required for ZFP57 binding in mouse ESCs. This finding is consistent with data recently obtained by Strogantsev *et al*. (38), who used a different cellular system (*M. m. castaneus* x *M. m. domesticus*). Together, the two studies provide the first comprehensive analysis of ZFP57 binding in the genomes of three different mouse sub-species, *M. m. domesticus, M. m. castaneus* and *M. m. molossinus*. The combined data demonstrate a high evolutionary conservation of ZFP57 binding at ICRs and frequent divergences else where in the genome, possibly underlying gene expression differences (Supplementary Table S6). Given that the three subspecies are thought to be less than 0.5 million years apart in evolutionary terms (34), this indicates that non-ICR ZFP57 sites undergo rapid evolution in mice, or that they show considerable polymorphism within mouse populations, which would then translates into differences between the inbred lines. Although the studies so far are too limited to draw firm conclusions, our data suggest that ZFP57 sites evolve fast and contribute to gene expression and phenotype changes during evolution.

The presence of SNPs in the TGCCGC motif only explained one third of the cases of strain-specific ZFP57 binding. Our study explored the possibility that ZFP57 also bound variants of this canonical motif. Consistent with this hypothesis, several SNPs were found in sequences diverging at only one position from the TGCCGC hexanucleotide. In particular, one (GGCCGC) of these sequences was demonstrated to bind ZFP57 *in vitro* if methylated. Interestingly, in about 10% of the ZFP57 ChIP-seq peaks including the one corresponding to the *Commd1* ICR, this motif variant replaces the more common TGCCGC. Other SNPs may possibly indirectly affect ZFP57 binding, by interfering with the binding of other factors competing with ZFP57 and/or altering the chromatin structure of the locus.

We have shown previously that ZFP57 is necessary for maintaining H3K9me3 and CpG methylation at ICRs (22). We now demonstrate that H3K9me3 co-localizes with ZFP57 at three loci with strain-specific ZFP57 binding, indicating that genetic differences in the ZFP57 binding motifs may result in differential maintenance of repressive epigenetic marks in ESCs. At another three sites, however, mono-allelic ZFP57 binding co-existed with bi-allelic H3K9me3 enrichment, indicating that factors independent of ZFP57 binding control H3K9me3 deposition at non-ICR sites as well. On the other hand, no allelic difference in the level of DNA methylation was found at three non-ICR loci showing strain-specific ZFP57 binding. Differently from ICRs, mono-allelic binding of ZFP57 at these sites is not determined by methylation but DNA sequence differences on the maternal and paternal chromosomes. In apparent contrast with this finding, the presence of differential methylation at some non-ICR loci with strain-specific binding was reported by Strogantsev *et al*. (38). This discrepancy may be ascribed to different ESC culture conditions or heterogeneity in the ZFP57 control of DNA methylation maintenance at non-ICR sites.

ICRs have the unique property of maintaining the DNA-methylated and the unmethylated status on the opposite parental alleles. It was previously shown that CTCF binding is necessary to maintain the maternal *H19* ICR non-methylated in mouse embryos and that the CTCF target site was required to prevent *de novo* methylation of this sequence in cultured ESCs (39,40). We found that a 477 bp fragment of the *Snrpn/Snurf* ICR is sufficient to maintain the original methylated or unmethylated status after introduction into ESC genome at an ectopic position and culturing for at least 15 cell divisions. This faithful somatic maintenance recapitulates the epigenetic properties of the endogenous ICR. The capacity of maintaining the methylated status was dependent on the presence of ZFP57 and its binding to three target sequence motifs. Significantly, the effect of motif mutations was most prominent when the transgenes were integrated into the β*-Globin* locus by homologous recombination, suggesting that the residual methylation of the randomly integrated mutant fragments in the *Zfp57* -/- ESCs was caused by spreading from flanking genomic loci. The analysis of the single motif mutations demonstrated that one of the motif sequences had a more relevant role than the others in the DNA methylation maintenance, indicating the existence of a hierarchy in the ZFP57 binding motifs. Finally, the 477 bp transgene included the *Snrpn/Snurf* minimal promoter that is part of an 1.2 kb transgene demonstrated to be sufficient to establish differential methylation, mono-allelic expression and asynchronous replication in the mouse (41,42). The different effect of the mutations on CpG methylation observed at ICR- versus non-ICR binding sites may possibly be explained by the CpG-rich and promoter-like characteristics of the former loci.

ZFP57 binding is generally associated with the presence of multiple motif sequences (22). We have now observed that closely spaced motifs (two or more [TG]GCCGC sequences less than 38 bp apart) are particularly frequent (40% of all peaks and 61% of those with two or more motifs) in the genomic regions covered by ZFP57 peaks. The functional relevance of these motif clusters is indicated by the observation that they are present at all ICRs and that their mutation usually leads to loss of ZFP57 binding. It is possible that interaction between closely located binding sites may synergistically increase the binding affinity for ZFP57. If we also take into consideration that many of the motif clusters that do not bind ZFP57 are present in non-methylated CpG-rich regions, our results significantly improve the structural definition of the ICRs and other ZFP57 binding sites and increase our understanding on the effect of DNA sequence changes on gene expression and disease phenotypes.

## ACCESSION NUMBER

Data for the ZFP57 and KAP1 Chip-seq in BJ1 and JB1 cell lines have been deposited in the GEO database with accession number GSE74757.

## SUPPLEMENTARY DATA

Supplementary Data are available at NAR Online.

SUPPLEMENTARY DATA
