# Supplementary material for: ZFP57 recognizes multiple and closely spaced sequence motif variants to maintain repressive epigenetic marks in mouse embryonic stem cells
Source: Nucleic Acids Res. 2015 Oct 19;44(3):1118–32. doi: 10.1093/nar/gkv1059 (PMC4756812; doi:10.1093/nar/gkv1059)
Supplement: SUPPLEMENTARY DATA [file supp_44_3_1118__index.html]

ZFP57 recognizes multiple and closely spaced sequence motif variants to maintain repressive epigenetic marks in mouse embryonic stem cells — ZFP57 recognizes multiple and closely spaced sequence motif variants to maintain repressive epigenetic marks in mouse embryonic stem cells — SUPPLEMENTARY DATA 

# ZFP57 recognizes multiple and closely spaced sequence motif variants to maintain repressive epigenetic marks in mouse embryonic stem cells

## SUPPLEMENTARY DATA

- SUPPLEMENTARY DATA
- SUPPLEMENTARY DATA
- SUPPLEMENTARY DATA
- SUPPLEMENTARY DATA
- SUPPLEMENTARY DATA
- SUPPLEMENTARY DATA
- SUPPLEMENTARY DATA
- SUPPLEMENTARY DATA
- SUPPLEMENTARY DATA
- SUPPLEMENTARY DATA
- SUPPLEMENTARY DATA
- SUPPLEMENTARY DATA
- SUPPLEMENTARY DATA
- SUPPLEMENTARY DATA
