# Supplementary material for: ZFP57 recognizes multiple and closely spaced sequence motif variants to maintain repressive epigenetic marks in mouse embryonic stem cells
Source: Nucleic Acids Res. 2015 Oct 19;44(3):1118–32. doi: 10.1093/nar/gkv1059 (PMC4756812; doi:10.1093/nar/gkv1059)
Supplement: SUPPLEMENTARY DATA [file supp_gkv1059_nar-02093-m-2015-File008.docx]

**SUPPLEMENTARY FIGURES AND TABLES LEGENDS**

**Supplementary Figure 1** (related to Figure 1). Allele-specific expression analysis of *Zp3r* in JB1 and BJ1 ESCs. DNA sequencing electropherograms obtained from PCR products of DNA and cDNA regions containing a SNP. The SNP is reported with the JF1 allele in bold, and shaded in grey in the electropherogram. Note the preferential expression of the B6 allele in both cell lines.

**Supplementary Figure 2** (related to Figure 1). Validation of allele-specific ZFP57 binding by locus-specific ChIP and sequencing of the immunoprecipitated DNA in BJ1 and JB1 ESCs

**Supplementary Figure 3** (related to Figure 3). Allele-specific H3K9me3- and ZFP57-ChIP analyses at loci with mono-allelic ZFP57 binding and bi-allelic H3K9me3 enrichment.

**Supplementary Figure 4.** DNA methylation analysis of selected non-ICR ZFP57 target sites in JB1 ESCs by bisulfite sequencing of isolated clones.

**Supplementary Figure 5.** Allele-specific expression in JB1 and BJ1 ESCs. DNA and cDNA sequencing electropherograms show the contribution of the B6 and JF1 alleles of genes located close to strain-specific ZFP57 binding sites. Examples of two genes with expression skewed towards the B6 allele (the one bound by ZFP57) (A) and two genes with equivalent expression from the B6 and JF1 alleles (B). SNPs are reported as in Suppl. Fig. 1.

**Supplementary Figure 6** (related to Figure 6). UCSC screen shot showing ZFP57 and KAP1 binding at the *Commd1* ICR associated with two overlapping and inverted copies of the GGCCGC sequence motif variant, as determined by ChIPseq.

**Supplementary Figure 7** (related to Figure 6). Empirical cumulative distribution of the distances separating two [TG]GCCGC (orange) or two TGCCGC (purple) sequences in the genomic regions covered by ZFP57 peaks.

**Supplementary Table 1**. Primers and EMSA probes. The .docx file contains information on primer and EMSA probe sequences.

**Supplementary Table 2**. JB1 and BJ1 ZFP57 peaks. The .xlsx file contains information on the 545 ZFP57 peaks coinciding with KAP1 binding sites and present in both reciprocal cell lines. For each peak is indicated: the chromosomal coordinates (assembly NCBI37/mm9), the closest gene (gene symbol), the number of occurrences of analyzed motif (TGCCGC, GGCCGC and [TG]GCCGC) and the number of informative reads (IR) and allele-specific reads (ASR) for each hybrid cell line, mapped with or without mismatches.

**Supplementary Table 3**. Informative ZFP57 peaks. The .xlsx file contains information on the 151 ZFP57 informative peaks. For each peak is indicated: the peak ID, the chromosomal coordinates (assembly NCBI37/mm9), the percentage of allele-specific reads *x* for each hybrids cell line, the allelic score (S) and the type of allelicity.

**Supplementary Table 4**. SNPs and sequence strings overlapping ZFP57 peaks. SNPs in ZFP57 peaks. This .xlsx sheet contains information on the SNPs overlapping the 151 informative ZFP57 peaks. For each SNP is indicated: the SNP ID, the peak ID (as in Suppl. Table 2), the chromosomal coordinates (assembly NCBI37/mm9), the type of allelicity of the peak containing the SNP, the base changes between B6 and JF1 genome, the insertions-deletions between B6 and JF1 genome (the position is related to the sequence ± 5 bp surrounding the SNP), the B6 and JF1 sequence string surrounding ± 5 bp the SNP. Permissive and non-permissive alleles. In the second sheet, is indicated: the SNP ID (as in Suppl. Table 2), the permissive and the non permissive sequence strings surrounding ± 5 bp the SNPs overlapping the genotype-specific ZFP57 binding sites.

**Supplementary Table 5**. Motif distances under ZFP57 peaks and [TG]GCCGC

motif clusters. The first .xlsx sheet contains information on the distances between TGCCGC sequences under ZFP57 peaks. The second .xlsx sheet contains information on the distances between [TG]GCCGC motif under ZFP57 peaks. The third .xlsx sheet contains information on the chromosomal coordinates of [TG]GCCGC motif clusters of at least two motif sequences less than 38 bp apart and occurrences of [TG]GCCGC in each cluster.

**Supplementary Table 6**.

Strain-specific ZFP57 binding sites identified in this and Strogantsev’s studies. Cast, *M. m. castaneus*.
