# Supplementary figures and images for: ZFP57 recognizes multiple and closely spaced sequence motif variants to maintain repressive epigenetic marks in mouse embryonic stem cells
Source: Nucleic Acids Res. 2015 Oct 19;44(3):1118–32. doi: 10.1093/nar/gkv1059 (PMC4756812; doi:10.1093/nar/gkv1059)

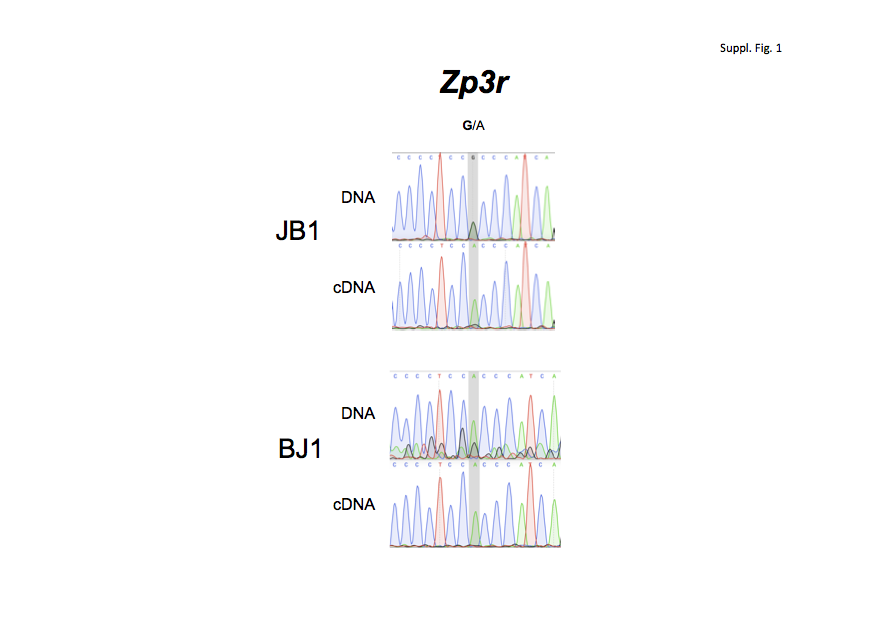

Supplement: SUPPLEMENTARY DATA [file supp_gkv1059_nar-02093-m-2015-File009.tif]

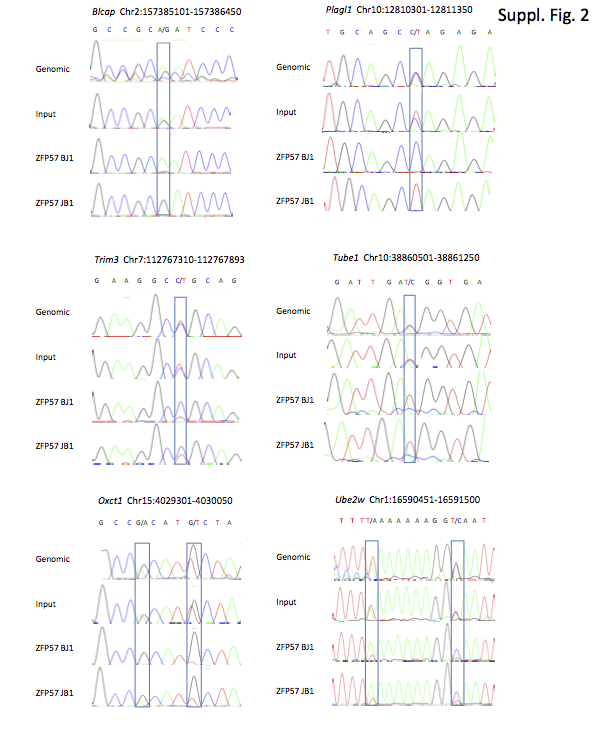

Supplement: SUPPLEMENTARY DATA [file supp_gkv1059_nar-02093-m-2015-File010.tif]

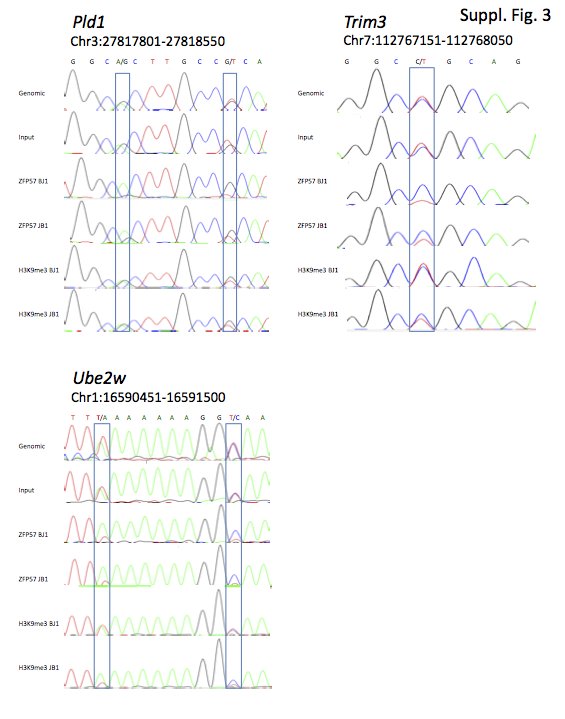

Supplement: SUPPLEMENTARY DATA [file supp_gkv1059_nar-02093-m-2015-File011.tif]

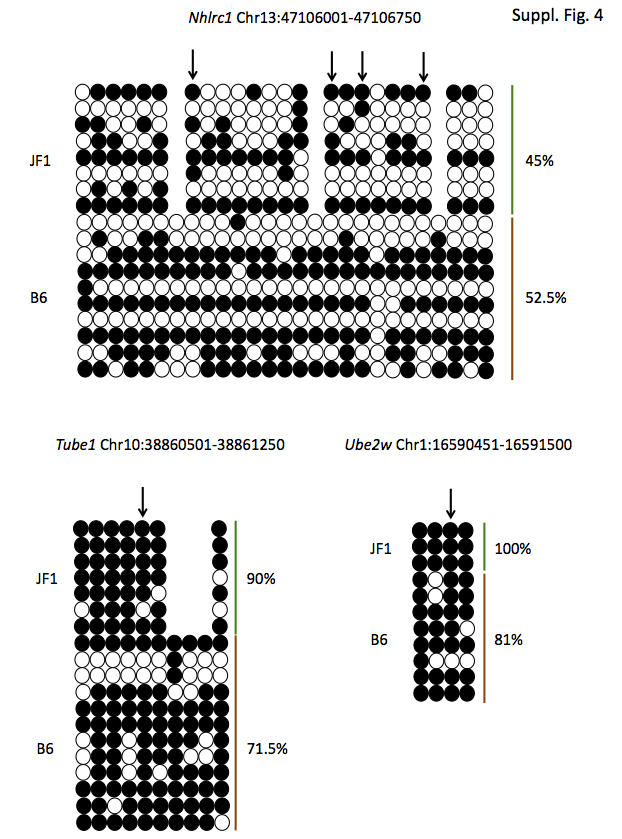

Supplement: SUPPLEMENTARY DATA [file supp_gkv1059_nar-02093-m-2015-File012.tif]

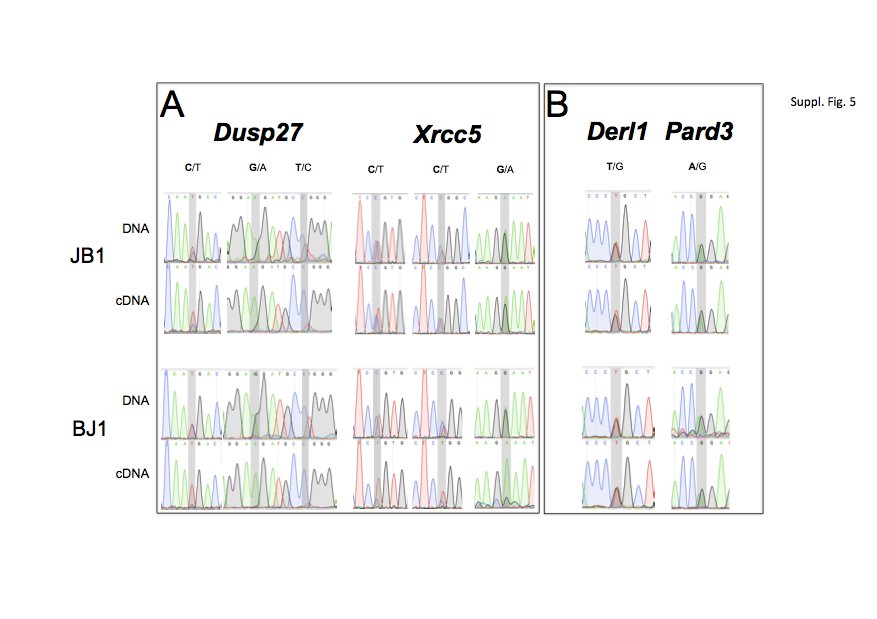

Supplement: SUPPLEMENTARY DATA [file supp_gkv1059_nar-02093-m-2015-File013.tif]

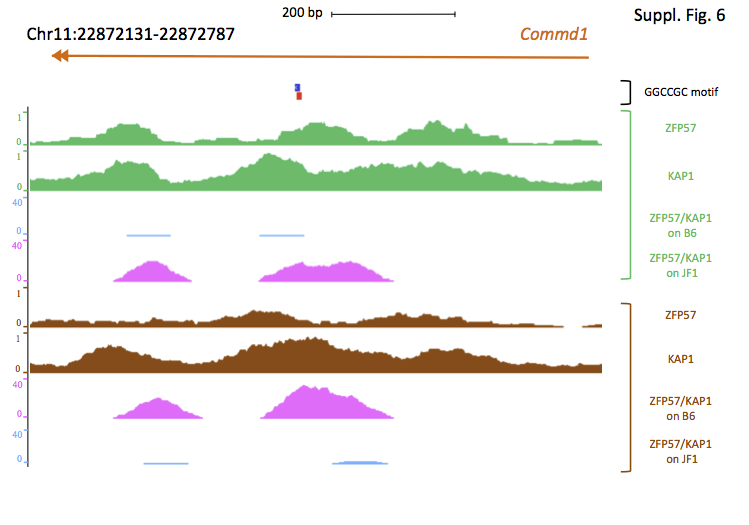

Supplement: SUPPLEMENTARY DATA [file supp_gkv1059_nar-02093-m-2015-File014.tif]

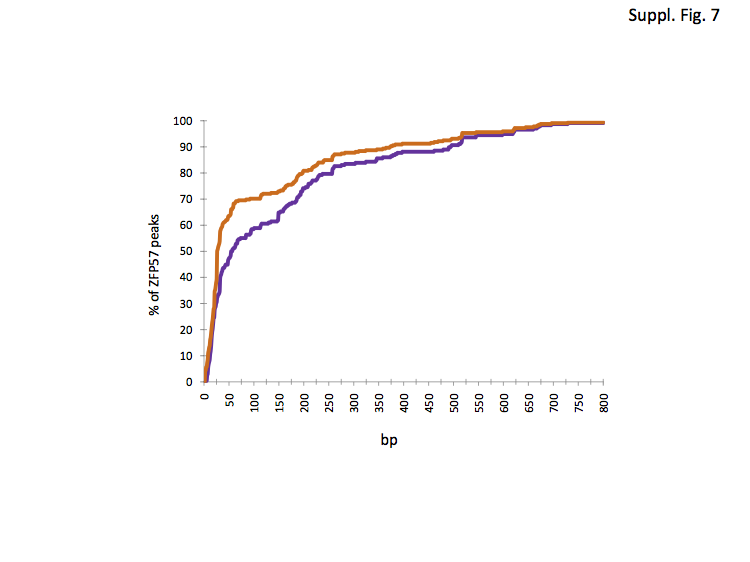

Supplement: SUPPLEMENTARY DATA [file supp_gkv1059_nar-02093-m-2015-File015.tif]
